# Image testing in psychiatrics: a bibliografic review

**DOI:** 10.1192/j.eurpsy.2022.1645

**Published:** 2022-09-01

**Authors:** M.D.C. Molina Liétor, I. Cuevas Iñiguez, A. Sanz Giancola, C. Alvarez Garcia

**Affiliations:** 1 Hospital Universitario Príncipe de Asturias, Psiquiatría, Alcalá de Henares, Spain; 2 Hospital Principe de Asturias, Psiquiatría, Alcala de Henares, Spain; 3 Hospital Universitario Príncipe de Asturias, Psychiatry, Alcalá de Henares, Spain

**Keywords:** Neuroimaging, Psychoradiology

## Abstract

**Introduction:**

Psychoradiology is a term that describes a growing interest in relating psychiatry and radiological images, proposing a radiological approach in the management of major psychiatric illnesses. This includes the diagnosis, the planning of the treatment and the study of the clinical course.

**Objectives:**

The objective of this communication is to review the current status of the importance and indications of neuroimaging tests in psychiatry.

**Methods:**

A literature review has been carried out to review this issue.

**Results:**

In schizophrenia, longitudinal studies have been carried out that compare the anatomical structures between a first psychotic episode and in a chronic state, locating regional changes that progress as the disease does. Anatomical alterations have also been detected among patients with a predominance of positive symptoms or negative symptoms. Although more and more studies demonstrate a certain common genetic and radiological basis between bipolar disorder and schizophrenia, imaging techniques can also show specific findings that differentiate one pathology from the other. The neuroimaging tests used in psychiatry are: • Brain CT, recommended when a first psychotic episode is suspected. • MRI: recommended in processes of cognitive deterioration, to evaluate white matter and for pregnant patients. It is also recommended to evaluate injuries that could have a poor prognosis with the application of electroconvulsive therapy. • Functional tests (PET and SPECT) are often used to screen some types of dementia such as Alzheimer’s or for research.

**Conclusions:**

New advances and knowledge in psychiatry and radiology must be integrated for better clinical practice.

**Disclosure:**

No significant relationships.

